# Biomedical engineering and interdisciplinary research in shaping tomorrow’s medicine

**DOI:** 10.1016/j.isci.2025.112884

**Published:** 2025-07-02

**Authors:** Angela Chen, Carly Harris, Cheng-Kui Qu

**Affiliations:** 1 Department of Pediatrics, Emory University School of Medicine, Aflac Cancer & Blood Disorders Center, Winship Cancer Institute, Children’s Healthcare of Atlanta, Atlanta, Georgia, USA

## Abstract

Dr. Wilbur Lam merges pediatrics, biomedical engineering (BME), and innovation to advance patient care through interdisciplinary research. From developing a non-invasive smartphone app for anemia patients to leading national COVID-19 diagnostic efforts, Dr. Lam exemplifies how interdisciplinary collaboration can transform patient care. Blending passion, practicality, and scientific creativity, Dr. Lam inspires the next generation to bridge diverse fields in pursuit of accessible, life-changing medical solutions. His work sets a new standard for translational research and innovation in medicine.

**Figure:**
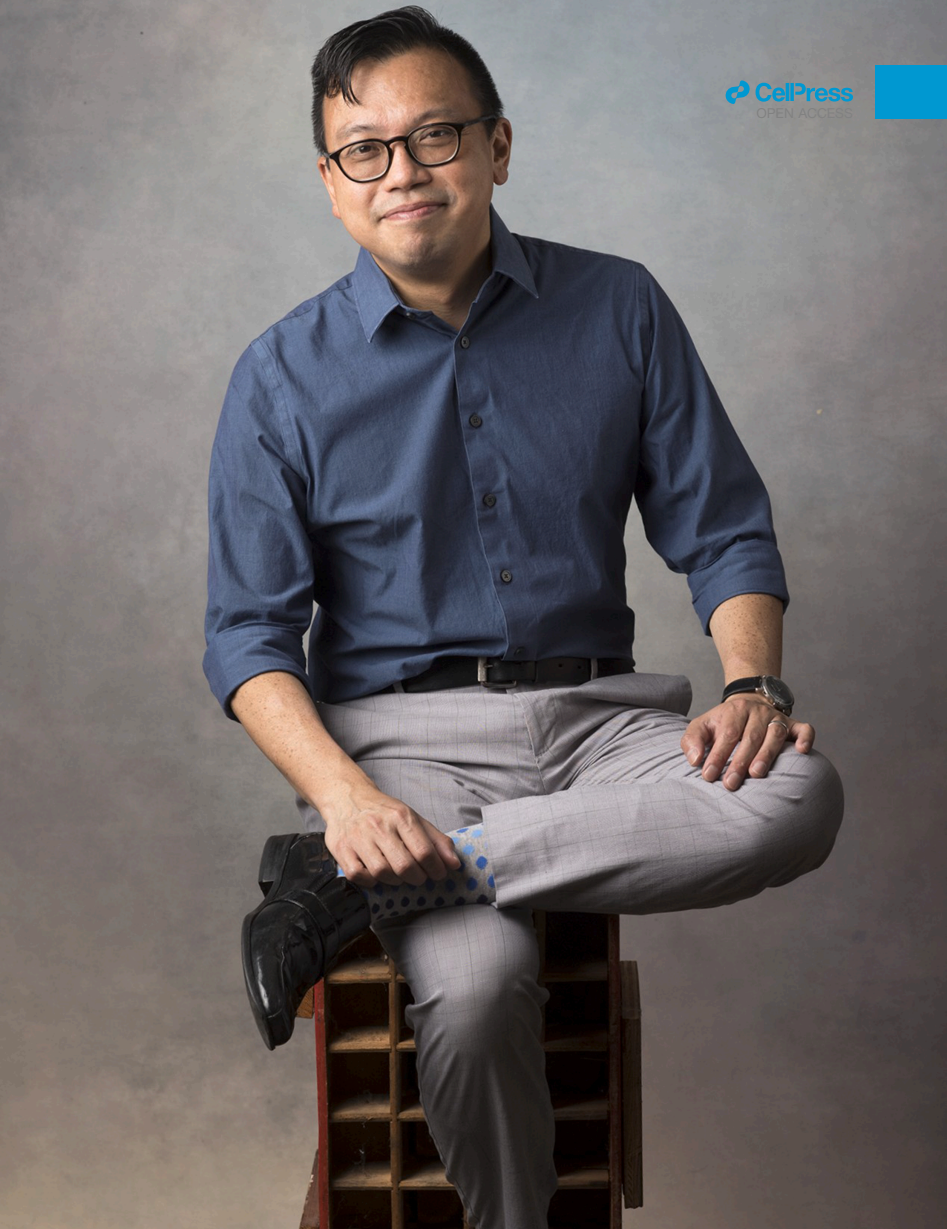
Above image: Headshot of Dr. Wilbur Lam

Physician-scientists like Dr. Lam are investing great effort into expanding our knowledge of hematological diseases, investigating the fundamental mechanisms of blood disorders, and exploring new treatments and applications to improve patient care.

It’s the combination of interests, combined with the opportunities that I had. It’s a combination of personal passion and practical feasibility.

That is how I define scientific creativity—it is the capability to strike that balance where you want to do something that is super cutting edge but not too cutting edge that it will yield higher risk.

However, true innovation comes when you bring in people who superficially don’t make sense together at all.

## Main text

Alan is a patient with chronic anemia who needs regular blood transfusions to avoid dangerously low hemoglobin levels. When Alan’s hemoglobin level gets too low, he experiences a myriad of symptoms, such as dizziness, fatigue, lightheadedness, and shortness of breath. To prevent this, he can simply take a picture of his fingernail with his smartphone. The app will use an image-based algorithm to identify Alan’s current hemoglobin level and accurately predict when he will need his next transfusion. While Alan is a fictional patient, many anemia patients like him rely on this life-changing smartphone app, which was developed in 2018 by a team of scientists led by physician-scientist Dr. Wilbur Lam. Dr. Lam is not only a pediatric physician-scientist but also Vice Provost for Entrepreneurship at Emory University, Director of Advancement of Diagnostics for a Just Society Center, Associate Dean of Innovation at Emory University School of Medicine, and Vice Chair of Innovation and Technology Research at Emory University Department of Pediatrics. The app that he and his team have created allows patients like Alan to monitor their diseases at home and adjust their therapeutic plans promptly so that potential side effects and complications can be reduced.^1^

Hematological diseases, including anemia, leukemia, lymphoma, and myeloma, are pressing health concerns that require greater awareness due to their early onset, rapid progression, and high mortality rates. According to the Leukemia and Lymphoma Society, one person in the US is diagnosed with blood cancer (leukemia, lymphoma, or myeloma) every three minutes.^2^ The American Cancer Society estimates that 66,890 people in the US will be diagnosed with leukemia in 2025, and 23,540 people with leukemia will die in 2025.^3^ Physician-scientists like Dr. Lam are investing great effort into expanding our knowledge of hematological diseases, investigating the fundamental mechanisms of blood disorders, and exploring new treatments and applications to improve patient care.

It is our pleasure to interview Dr. Lam, who leads a laboratory that serves as a unique “one-stop shop” for developing cutting-edge micro/nanotechnologies and mobile health platforms to study blood diseases and cancer. Dr. Lam and his team apply engineering technologies to cellular biology research, generating meaningful and groundbreaking results.^4^ By integrating concepts from biology, physics, and engineering, scientists like Dr. Lam can uncover the underlying mechanisms of a cell’s behavior from a brand-new perspective. Dr. Lam’s “basement to bench to bedside” approach translates biology benchwork conclusions into real-world scenarios, allowing his lab to invent and bring new technologies to the patient’s bedside with the aim of improving patient care and treatment. Dr. Lam’s interdisciplinary research approach is transforming the biomedical field, and he is sure to serve as an inspiration to many.

### Inspiration and impact

#### How did you start to work in biomedical engineering: Did you train in this area, or are your research interests aligned with the developing field?

Throughout my career, I have pursued projects that interest me. However, I have stepped into the field not just because of my passions but also because the projects I have had in the past led me there. And that is how I view things from a career perspective, evaluating opportunities in terms of whether a project is worth the time and effort of both myself and my team.

I entered the medical field at an early age—I was accepted into medical school when I was eighteen years old through a BS/MD program at the Baylor College of Medicine. After I graduated from college and went to medical school, I still wasn’t sure about what I wanted to pursue. So, instead of just asking myself what I wanted to do, I also asked myself what I didn’t want to do. I realized that I liked to work with children, which ultimately led me to decide to become a pediatrician. Later, as my training progressed, I was exposed to different fields of research, outside of pediatrics. Through these opportunities, I became passionate about technology and engineering development, so I pursued and earned a PhD in Bioengineering. It was a long journey, but I knew that my education would allow me to make an impact.

In every project you embark on, it is important to make sure that to the best of your capability, there is some likelihood that you’ll make an impact. Impact can be defined in many ways, but you should strive to achieve a certain number of milestones that should bring forth success for a project. That is how I ended up in the career path that I took. I was already a pediatrician, and I saw that there was a need for new biomedical technologies in the care and research of child health. So, then I also thought about what it was that I was intrinsically interested in—later I found out that it is blood and cancer. It’s the combination of interests, combined with the opportunities that I had. It’s a combination of personal passion and practical feasibility. I realized that to pursue my passion, I both desired and needed to achieve a certain degree of success with it.

#### What does a typical workday look like for you, and how do you balance multiple roles to manage your time effectively?

I do not have a typical day—every day is full of surprises and different for me. I hold several different roles, not only as a physician-scientist but also as an administrator. I am able to achieve success in my roles because I work with the best teams possible. I’m blessed to have a team of trustworthy individuals in each of my roles, allowing me to delegate tasks so that I don’t have to attend every meeting or make every decision. For example, the brilliant senior researchers in my laboratory manage the day-to-day operations and make sure the lab is running smoothly, even when I am not physically present. Similarly, in my administrative roles, I also work with a great team. It is important to have reliable teams that I can trust and delegate tasks to.

#### What inspires new discoveries for you? What is your thought process when developing each new experiment?

We try to think of ways to do things differently. To me, discovery is a combination of having two things that are different enough that there are not many people thinking this way and balancing them. But at the same time, it must be grounded in a scientific rationale. That is how I define scientific creativity—it is the capability to strike that balance where you want to do something that is super cutting edge, but not too cutting edge that it will yield higher risk. You must always balance feasibility with risk, and that is how we start new projects or proposals.

My lab and I work to incorporate this philosophy into all of our projects, such as our work culturing endothelial cells in microvascular-sized microchannels for *in vitro* study of cell interactions.^5^^,^^6^ This new technique we discovered allowed us to study blood diseases very accurately through the replication of many physiological factors. Our strength is being able to think differently, and that allows us to achieve success in endeavors like this.

### Innovation

#### In the future, how do you think your innovations and new findings such as the smartphone application for anemia patients can be utilized in patient care, especially with patients suffering from blood disorders?

I dislike jumping on bandwagons. So, when there’s a clinical problem in front of us that we’re trying to tackle, we always think about what the major shift is in the field right now. And that’s exactly the direction we want to avoid. Or, if we must take that direction, we look for a slightly different angle from the mainstream.

When it comes to blood disorders and cancer, we will think, “Which disease are we trying to attack? What aspects of the clinical problems are we trying to solve? What has been done? What is currently being done? What is the most exciting thing that the field is moving toward? When developing a solution, do we want to go either next to it, or approach to it?” It’s important to think about issues in novel ways to find novel solutions.

One exciting innovation I co-founded is an FDA-cleared smartphone app that allows anemia patients to monitor their hemoglobin levels. This point-of-care app is a non-invasive option for detecting anemia by analyzing pictures of a patient’s fingernail color. It facilitates self-management by patients with chronic anemia, possibly reducing complications.^1^ In this case, it was important to merge technology and biomedical research to best help our patients, and this interdisciplinary approach can certainly be applied to many other projects.

#### What BME concept(s) do you think will become more prevalent in upcoming years?

I think the concept of non-invasive monitoring of physiological signals will become increasingly important, with new advancements and fields, such as biophysics in nature, related to wearable technologies. There is increasing potential for many medical technologies to be used at home with more accessibility. Within this, I’m especially excited about the concept of being able to learn about a person’s health without having to invasively obtain tissue or draw blood. The less invasive the technology, the higher the likelihood that it can be used at home to monitor a disease.

I’ve been involved with similar research in the past, like with the hemoglobin app for anemia patients I mentioned earlier. I’ve also contributed to other similar projects focusing on point-of-care health such as a smartphone-based technology that can assess a patient’s acute abdominal pain to a similar degree of accuracy as a physician.^11^ Inventions like these that shift healthcare into the home make healthcare less expensive and more accessible, which are critical to improving health in underserved areas. My lab strives to make healthcare more accessible through these concepts, so I can see us potentially continuing to pursue non-invasive monitoring technologies.

### Interdisciplinary research

#### How do you view the importance of integrating other fields into basic science research, so that an improvement or advancement in the field can be made?

I think that highlights how we think. One potential way to be scientifically creative is to take a problem, take a typical approach, and then marry it with a seemingly extremely disparate approach. This is often the path toward innovation, where you force yourself to think of the problem through lenses that are completely atypical of what the current state is for that field. So, it’s more about how we bring in other fields to tackle a problem in a specific field.

This approach is not just for us scientists but for human creativity in general. This is part of the reason why people talk about the need for diverse teams. For example, when you’re dealing with a problem, like cancer, you can look at the problem from a biological perspective. But what if you look at things from a physical perspective? And let’s think about it even more creatively, right? What if we look at things, not just from a physical perspective, but we start to think of things by how they are related to other aspects of science? What if we incorporate the field of physical energy or social sciences? Sometimes social scientists have tools, like analytical software, that could be leveraged to solve biomedical problems. For example, there may be statistical tools that social scientists use for data analysis that could be very useful for us in biomedical science. We just don’t know about them, because it’s not inherent. It’s not used customarily in our field. Even data analysis can be used to look at things in a different way.

#### How can we as a scientific community continue to expand interdisciplinary work to benefit more patients and improve patient care?

The major thing is how to bring in communities that don’t normally collaborate, to solve big problems together. Right now, we usually bring in folks that share some commonalities. However, true innovation comes when you bring in people who superficially don’t make sense together at all. But once you come together and talk, you could find that some links could be very exciting and can bring these two fields together to address a common problem.

To continue to expand interdisciplinary work, we need to continue to seek these links between fields, and hunt for unexpected connections. In doing this, we can continue to find innovative solutions and improve the patient-care landscape. Interdisciplinary work in the medical community does not need to be limited to just two scientific fields—it can also be an intersection of business and science. Great business strategies combined with new scientific findings can allow us to manufacture more medical products at a lower cost, making them more accessible to patients.

#### As the Vice Provost for Entrepreneurship at Emory University, how should we promote interdisciplinary BME and biomedical research from your leadership perspective?

I have a few leadership roles that I’m involved in, and all these roles are similar in that they promote interdisciplinary research. To promote interdisciplinary biomedical engineering (BME) and biomedical research, we first must ensure that there are ways to think about how new technologies can be commercialized. We don’t always want to think about this, but it is the only way to have a true impact, especially when it comes to patient care. If you want to ensure that what you are doing can impact people around the world, it needs the ability to be commercialized. It is not about trying to make money but more about scalability and improving the probability that this invention will touch as many patients’ lives as possible.

This concept was especially important during the COVID-19 pandemic when I was the principal investigator of the Test Verification Center for the NIH RADx initiative to assess COVID-19 diagnostics.^5^^,^^6^^,^^7^^,^^8^^,^^9^ In this role, my goal was to assess the accuracy of COVID-19 tests and to ensure they were easy to use and manufacture. Ensuring that a test is accurate and reliable is very important but is only truly useful if that test can be purchased and used by the average patient. The instructions must be clear and easy to understand, and the manufacturing process must be simple enough that the test can be done on a larger scale at a lower cost, to ensure it can reach the greatest number of patients possible.

Business and science create an interdisciplinary field of their own. When creating new medical products, these technologies should be easily commercialized so they can be accessible to all. This idea is also present in technologies like stem cells or cell replacement therapies, where scientists have been using microcarriers to increase the production of stem cells for large-scale commercialization.^10^ This conversion of basic scientific research into large-scale commercial products enables the treatment of more patients by making discoveries more accessible, and therefore more effective in improving medical care in both clinical and home settings.

#### What suggestion would you give to a young scientist interested in interdisciplinary research?

Firstly, I would recommend looking into yourself and figuring out what fields you’re most passionate about, which don’t necessarily need to be superficially related at first glance. Once you know what you’re passionate about, do research into the fields and see what links there are between the fields that people haven’t been thinking about. If you can make a scientific rationale that marries these two fields together, you will find the cross-disciplinary field worth researching. “Interdisciplinary training” is not the best wording for this because that means you are doing it in between. Instead, “cross-disciplinary training” is a better phrase as you learn a field first, then you learn another field, and then you combine the two, crossing between them. And if you can bring two disparate fields together, you can start to think of some interesting things in the middle, that likely very few people are thinking about. And that’s how you attack those problems and “solve problems in an innovative way”, as they say.

## Acknowledgments

The authors acknowledge support from National Institutes of Health (HL162725, HL130995, and CA282579).
